# Do diets with higher carbon footprints increase the risk of mortality? A population-based simulation study using self-selected diets from the USA

**DOI:** 10.1017/S1368980022000830

**Published:** 2022-08

**Authors:** Benjamin D Pollock, Amelia M Willits-Smith, Martin C Heller, Lydia A Bazzano, Donald Rose

**Affiliations:** 1 School of Public Health and Tropical Medicine, Tulane University, 1440 Canal Street, Suite 2200, New Orleans, LA 70112, USA; 2 Robert D. and Patricia E. Kern Center for the Science of Health Care Delivery, Mayo Clinic, Jacksonville, FL, USA; 3 Center for Sustainable Systems, School for Environment and Sustainability, University of Michigan, Ann Arbor, MI, USA

**Keywords:** Carbon footprint, CVD, Dietary guidance, Mortality risk, National Health and Examination Survey, Preventable Risk Integrated Model, United States

## Abstract

**Objective:**

Are diets with a greater environmental impact less healthy? This is a key question for nutrition policy, but previous research does not provide a clear answer. To address this, our objective here was to test whether American diets with the highest carbon footprints predicted greater population-level mortality from diet-related chronic disease than those with the lowest.

**Design:**

Baseline dietary recall data were combined with a database of greenhouse gases emitted in the production of foods to estimate a carbon footprint for each diet. Diets were ranked on their carbon footprints and those in the highest and lowest quintiles were studied here. Preventable Risk Integrated Model (PRIME), an epidemiological modelling software, was used to assess CVD and cancer mortality for a simulated dietary change from the highest to the lowest impact diets. The diet–mortality relationships used by PRIME came from published meta-analyses of randomised controlled trials and prospective cohort studies.

**Setting:**

USA.

**Participants:**

Baseline diets came from adults (*n* 12 865) in the nationally representative 2005–2010 National Health and Nutrition Examination Survey.

**Results:**

A simulated change at the population level from the highest to the lowest carbon footprint diets resulted in 23 739 (95 % CI 20 349, 27 065) fewer annual deaths from CVD and cancer. This represents a 1·83 % (95 % CI 1·57 %, 2·08 %) decrease in total deaths. About 95 % of deaths averted were from CVD.

**Conclusions:**

Diets with the highest carbon footprints were associated with a greater risk of mortality than the lowest, suggesting that dietary guidance could incorporate sustainability information to reinforce health messaging.

Climate change is among the most pressing environmental problems^(1)^ and has serious consequences for human health^(2)^. Dietary choice is the nexus between environment and health, since human food systems contribute roughly one-third of greenhouse gases worldwide^(3)^ and poor diets contribute to chronic disease. Ample evidence indicates that diets with more red and processed meat increase the risk of mortality, while those with more vegetables and fruit are associated with decreased risks for cancer, CVD and mortality^(4)^. Plant-based diets, such as vegetarian or vegan diets, also have lower carbon footprints^(5)^. This is because the production of animal foods, particularly ruminant animals, is responsible for greater amounts of greenhouse gas emissions (GHGE) than is the production of plant foods. For example, the GHGE in the production of beef is 8–10 times that of chicken and around 20 times that of some legumes, nuts or seeds^(6,7)^.

Nevertheless, the links between diet, environment and health are not straightforward. For example, lower carbon footprint diets may have less meat, so they are likely to have lower levels of saturated fats. This was found in analysis of self-selected US diets^(8)^, as well as in an earlier systematic review of the literature^(9)^. However, such diets may also have lower levels of Fe, Ca or other micronutrients, which could be explained by fewer nutrient-dense animal foods in these diets and/or a greater concentration of ‘empty-calorie’ foods, such as sugars and oils that can be produced with relatively low GHGE^(8–10)^.

While most studies indicate that lower environmental impact diets lead to better health outcomes, some do not. Aleksandrowicz and colleagues reviewed studies which modelled health impacts from changes in typical Western diets to alternative sustainable diets^(11)^. Of the six cases which modelled all-cause mortality from these diet shifts, all showed mean reductions in mortality risk, but only three were statistically significant. They also found reductions in risks of diabetes, colorectal cancer and CHD with sustainable diet changes, but these were only statistically significant for diabetes among women and for colorectal cancer among both men and women^(11,12)^.

There have been very few studies on the relationship between diet, environment and health in the USA. Data from a large prospective study of Seventh-day Adventists in the USA and Canada showed that vegetarian diets were associated with lower GHGE as well as a lower all-cause mortality^(13)^. Hallstrom and colleagues modelled dietary change in the USA from a standard American diet to several healthy alternative diets^(14)^. They found reductions in both GHGE and the relative risk of CHD, colorectal cancer and type 2 diabetes. These are both important studies, but the Adventist study is not representative of the US population overall and the latter study is based on aggregate food availability data and generic recommendations. This leaves a notable gap in the existing literature – to our knowledge there have been no nationally representative studies on environmental and health outcomes in the USA that are based on individual self-selected diets.

We previously developed a distribution of diets ranked by GHGE from a nationally representative sample of US adults^(7,8)^. In this paper, we assessed the potential consequences of these diets for US population health by testing whether self-selected American diets with the highest GHGE predicted greater population-level CVD and cancer mortality than diets with the lowest GHGE.

## Methods

We utilised the Preventable Risk Integrated Model (PRIME) to simulate the annual cardiovascular and cancer deaths that could be averted by switching from self-selected diets that were highest in GHGE to those that were lowest in GHGE^(15)^. PRIME uses empirical data from user-specified baseline diets and cause-specific mortality along with diet–mortality relationships that come from published meta-analyses of randomised controlled trials and prospective cohort studies. These literature-based diet–mortality relationships are net effects after controlling for behavioural and demographic confounders.

Dietary data from a 24-h recall in a nationally representative sample of Americans aged 18–64 years (*n* 12 865) were obtained from the 2005–2010 National Health and Examination Survey (NHANES). Using our previously published Database of Food Impacts on the Environment for Linking to Diets (DataFIELD)^(7,8)^, we calculated the food-related GHGE from each individual NHANES diet and grouped them into sex-specific quintiles of GHGE per 1000 kilocalories. Dietary components for each individual were standardised to an energy intake appropriate to their age-sex group (see online supplementary material, Supplemental Table 1). The age- and sex-specific mean contents of diets in the highest (HiGHGE) and lowest (LoGHGE) quintiles of GHGE served as our test diets, which are shown in Table 1 for those 25–39 years of age, the largest demographic group in our study (see online supplementary material, Supplemental Table 2, which shows diets for all age groups). For baseline mortality, we used publicly available International Classification of Diseases (ICD-9) age- and sex-specific mortality tables (see online supplementary material, Supplemental Table 3, which lists these mortality counts) for the 2007 US population, the midpoint year for our study (see online supplementary material, Supplemental Table 4, which lists population numbers by age and sex)^(16)^.

**Table 1:** Model inputs from high and low carbon footprint diets, adults 25–39 years, 2005–2010 NHANES

Daily dietary components	HiGHGE diet		LoGHGE diet	
	Mean	sd	Mean	sd
**Males ages 25–39**				
Kilocalories†	2400		2400	
Grams of fruit	206	333	214	257
% consuming <1 fruit portion	66	46‡	50**	47‡
Grams of vegetables	221	177	188*	154
% consuming <1 vegetable portion	10	29‡	11	30‡
Grams of fibre	14·7	8·0	19·6***	10·6
Grams of salt	4·2	1·4	3·6***	1·3
% of kilocalories from fat	34·4	8·5	29·6***	8·5
% of kilocalories from saturated fat	12·1	3·7	8·5***	3·2
% of kilocalories from MUFA	13·1	3·7	10·7***	3·7
% of kilocalories from PUFA	5·7	2·4	8·0***	3·2
Milligrams of cholesterol	408	229	213***	140
**Females ages 25–39**				
Kilocalories†	1800		1800	
Grams of fruit	208	298	157	189
% consuming <1 fruit portion	49	50‡	51	49‡
Grams of vegetables	226	274	179*	141
% consuming <1 vegetable portion	9	29‡	14	34‡
Grams of fibre	13·7	8·2	15·8**	9·1
Grams of salt	3·4	1·2	2·7***	0·9
% of kilocalories from fat	35·2	9·8	30·5***	9·3
% of kilocalories from saturated fat	12·4	4·1	8·4***	3·0
% of kilocalories from MUFA	13·0	4·3	11·0***	4·3
% of kilocalories from PUFA	6·3	3·2	8·6***	3·7
Milligrams of cholesterol	274	170	134***	102

NHANES, National Health and Nutrition Examination Survey from the USA; HiGHGE and LoGHGE, highest and lowest quintiles of diets ranked on greenhouse gas emissions; sd, standard deviation; MUFA, monounsaturated fatty acids; PUFA, polyunsaturated fatty acids.

Mean values were significantly different from those of HiGHGE diet group: *P < 0·05, **P < 0·01, ***P < 0·001.

† Kilocalorie values for males and females are not mean values. Rather, they were assigned based on recommended values. Please see Supplemental Table 1 for details.

‡ The PRIME model does not use these sd (on percent consuming <1 portion of fruits or vegetables) as model inputs, but they are presented here for completeness.

In our PRIME model run, we estimated the annual cause-specific deaths averted if the US population’s average diet shifted from the HiGHGE diet to the LoGHGE diet. For this run, 95 % CI for deaths averted were calculated using a 10 000-iteration Monte Carlo simulation^(10)^. We also calculated the percentage of deaths averted for each cause, by dividing the PRIME results for deaths averted by the total number of baseline deaths in the midpoint year of our study. In PRIME simulations, averted deaths may be attributed to multiple causes, so percentages are approximate and may sum to more than 100 %.

To provide additional insights into the diets at the root of this analysis, we analysed mean consumption levels of protein-rich foods in both the Lo and HiGHGE diets. This included beef, pork, poultry, fish and seafood, eggs, dairy products, soya, legumes, and nuts and seeds. Differences between these mean levels of consumption were assessed using *t* tests. For comparison purposes, dairy consumption amounts were based on solids, that is, fats plus non-fat milk solids. The mean carbon intensity of these foods (kg CO_2_-eq per 100 g edible portion) was also assessed by averaging all available studies on these foods in DataFIELD.

## Results

Inputs for the PRIME simulation run for males and females, aged 25–39 years, are shown in Table 1 (and for the rest of the age groups in online supplementary material, Supplemental Table 2). For males, those with a diet that ranked in the lowest quintile of GHGE (i.e. LoGHGE) consumed, on average, 19·6 ± 10·6 g fibre, which was significantly greater (*P* < 0·05) than the 14·7 ± 8·0 g consumed by those males in the HiGHGE group. LoGHGE diets were lower in salt, saturated fats and monounsaturated fats, and higher in polyunsaturated fats than the HiGHGE diets. The LoGHGE diets were also lower in mean vegetable intakes than the HiGHGE diets. These patterns were the same for females, aged 25–39 years.

A simulated change from the HiGHGE diet to the LoGHGE diet resulted in 23 739 (95 % CI 20 349, 27 065) fewer annual deaths from CVD and cancer (Table 2). Most of these deaths averted (∼ 95 %) were from reductions in CVD rather than cancer, and within that category, the largest contributor to the reduction was from CHD.

**Table 2:** Cause-specific deaths averted by switching from high to low carbon footprint diets using the PRIME simulation

Cause of death	Deaths averted		Total deaths*	Percent of total deaths averted	
	Estimate of deaths averted (EDA)	95 % CI		EDA	95 % CI
**CVD** †	22 486	22 192, 22 780	648 440	3·47	3·42, 3·51
CHD	19 891	19 614, 20 167	406 277	4·90	4·83, 4·96
Stroke	2041	1953, 2130	135 679	1·50	1·44, 1·57
Heart failure	72	56, 89	56 547	0·13	0·10, 0·16
Aortic aneurysm	44	31, 57	12 977	0·34	0·24, 0·44
Rheumatic heart disease	5	1, 9	3197	0·15	0·02, 0·29
Hypertensive disease	434	394, 475	33 763	1·29	1·17, 1·41
**Cancer** †	1279	1209, 1349	212 334	0·60	0·57, 0·64
Lung	558	512, 604	158 751	0·35	0·32, 0·38
Colorectal	736	683, 789	53 583	1·37	1·27, 1·47
**Total deaths averted** †	23 739	20 349, 27 065	1 298 277	1·83	1·57, 2·08

PRIME, Preventable Risk Integrated Model.

95 % CI, the 95 % CI around the estimate of deaths averted.

* Total deaths in the USA for 2007, the mid-point year of our study, by cause of death.

† PRIME simulation does not specify that non-bolded sub-categories must sum to the exact bolded total. Averted deaths may be attributed to multiple causes. See reference^10^.

Total mortality for the midpoint year of our study was 1 298 277 deaths. Thus, the total simulated deaths averted for a population switch from the HiGHGE to the LoGHGE diet represent a decrease of 1·83 % (95 % CI 1·57 %, 2·08 %). The decline was much higher for CVD (3·47 %, 95 % CI 3·42 %, 3·51 %) than for cancer (0·60 %, 95 % CI 0·57 %, 0·64 %).

PRIME categorises deaths averted by broad dietary component – fats, salt, fibre and fruits/vegetables. In simulating a change from a Hi to a LoGHGE diet, changes in the fats group account for the largest amount of deaths averted at 17 203 (95 % CI 16 946, 17 460), followed by fibre (5799; 95 % CI 5649, 5948) and salt (2475; 95 % CI: 2378, 2573) (results not shown in table).

**Fig. 1:**
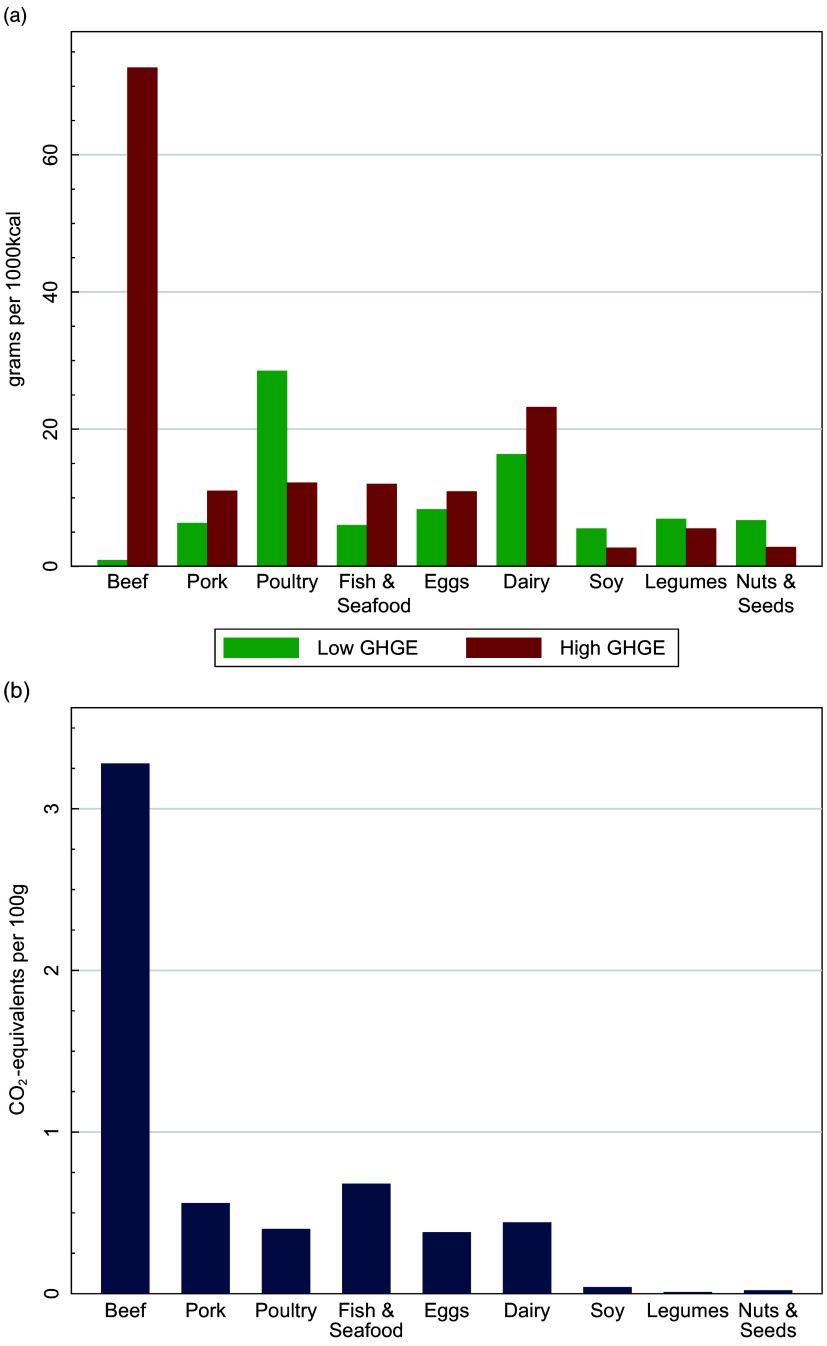
Protein-rich food consumption and emissions in the 2005–2010 National Health and Nutrition Examination Survey. (a) Difference in mean consumption (g/1000 kcal) between the lowest and highest quintile diets when ranked by greenhouse gas emissions (GHGE, kg CO_2_− eq/1000 kcal). All differences between the two groups of diets are statistically significant (*P* < 0·05). (b) Average greenhouse gas emissions (kg CO_2_− eq/100 g) of these foods (see reference^7^)

Figure 1a displays differences in the two diets in protein-rich foods, which might be driving the above results. Mean intakes of beef are many times higher in the HiGHGE diets than the LoGHGE diet. Mean intakes of other animal food sources, including pork, fish and seafood, eggs and dairy products, are also higher in the HiGHGE diet. On average, poultry, legumes, nuts and seeds, and soya are consumed in greater amounts in the LoGHGE diet. Differences in the GHGE intensities of these foods (kg CO_2_−eq/100 g) are presented in Fig. 1b. Beef has the greatest carbon intensity of any of the foods considered, followed by fish and seafood and the other animal-source foods.

## Discussion

Using a well-known and comprehensive diet–mortality model (PRIME), self-reported diets from the nationally representative NHANES and our novel environmental impact database, we found evidence that American diets in the highest quintile of GHGE are associated with a greater risk of mortality than American diets in the lowest quintile of GHGE. On a population level, a change from the HiGHGE to the LoGHGE diets would result in about 24 000 fewer deaths per year. This was about 2 % of total deaths in 2007, the midpoint year of our study. Although our analysis showed potential reductions across both domains of chronic disease mortality, most of the deaths averted, about 95 % of them, were due to declines in CVD.

What could be causing this simulated reduction in deaths? In addition to providing insights on the disease cause of death, our PRIME results indicated that the largest dietary cause was due to differences in dietary fats between the two diets. Dietary inputs from Table 1 show that the LoGHGE diets were higher in polyunsaturated fats and lower in saturated fats than the HiGHGE diets. This is consistent with the differences in the food composition of the two diets, shown in Fig. 1a. The LoGHGE diets had more plant protein foods and fewer animal protein foods than the HiGHGE diets, for all types of animal foods, except poultry. The differences in poultry and beef composition of the two diets are likely an important factor driving the simulated deaths as well as the differences in GHGE. Average poultry consumption in the LoGHGE diets is over twice that of the HiGHGE diets, while beef consumption is over 80 times higher in the HiGHGE diets. Our results indicated that the production of beef releases, on average, over 8 times the GHGE as poultry (Fig. 1). Literature values compiled by USDA indicate similar differences in saturated fat concentration between the two, with beef averaging 6·6 g of SFA per 100 g of edible portion compared with 0·8 g/100 g for chicken^(17)^. In sum, precisely where the two diets differ the most – in the beef and chicken content – are two foods that have dramatically different GHGE and saturated fat contents.

Our results are consistent with a previous study in which researchers used diet–disease relationships from meta-analyses to determine that decreasing population-average red meat consumption would lower risks of CHD as well as reduce expected emissions^(12)^. In another study from the United Kingdom, Scarborough and colleagues simulated several scenarios in which replacing meat and dairy with plant-based foods would result in significant reductions in both GHGE and mortality from cancer and CVD^(18)^. Although we did not explicitly simulate reductions in red meat or dairy, individuals in the LoGHGE group did have lower intakes of these foods than those in the HiGHGE group^(8)^, and this shows up in the difference in baseline saturated fats between the two groups (Table 1), as well as in Fig. 1a. A third study indicated that if the average UK diet shifted to meet WHO dietary guidelines, GHGE would be reduced by 17 % and the average life expectancy would increase by 8 months^(19)^.

Limited research in North America has linked dietary patterns to both emissions and mortality, although results from a highly selected population of Seventh-day Adventists are comparable to ours. In one such study, vegetarian diets were associated with a 9 % reduction in all-cause mortality and a 29 % reduction in GHGE compared with non-vegetarian diets among Adventists^(13)^. However, non-vegetarian diets from this religious group may not represent typical American diets. Hallstrom and colleagues used aggregate food availability data to represent the US diet and modelled a change from this to a healthier diet. They estimated this would result in a reduction in GHGE as well as decrease in risks of CHD, colorectal cancer and diabetes. Here, we did not design a healthier diet to be used in a normative way, we did not use food availability data to characterise the current American diet nor did we estimate relative risks of disease. But there is a common thread to our findings: a shift to a healthier diet would result in fewer chronic disease deaths and reduce GHGE from the food system. The cumulative dietary GHGE from the top quintile diets in our study represented 41 % of total dietary GHGE in the USA, five times that of the bottom quintile group, which accounted for just 8 % of the total^(8)^.

We characterised the LoGHGE diets in our study as healthier than the HiGHGE diets, based on our previous research using a measure of diet quality^(8)^. The Healthy Eating Index (HEI) is an overall diet quality score developed in the USA^(20)^. On a hundred point scale, the mean HEI score for the LoGHGE diets was 2·3 points (∼4·7 %) higher than for the HiGHGE diets. The research presented in this paper confirms the directionality of these findings using a different metric, that is, shifting from a HiGHGE to a LoGHGE diet would avert deaths from CVD and cancer. Although these climate friendlier diets are healthier, they are not healthy in an absolute sense. The mean HEI score of the LoGHGE diets was only 50·3 points out of 100^(8)^. So, there is much room for improvement across the spectrum of US diets.

Our approach used self-selected dietary data from a national sample. This allowed us to create post-hoc groups of representative diets based on GHGE, rather than the pre-defined dietary patterns used in other studies (e.g. vegetarian). Previously, we found that only 2 % of 2007–2010 NHANES respondents considered themselves to be vegetarians^(21)^, a bit lower than the 5 % figure seen in a 2018 national poll^(22)^. Thus, our results are representative of a broader cross-section of Americans. One limitation of our study, though, is that PRIME uses the simplifying assumption that all vegetables and all fruits are of equivalent nutritional value. Nevertheless, this generalised approach has utility at the population level for promoting a simple public health message to ‘eat more vegetables’. Second, we assumed no changes in physical activity, alcohol or smoking despite PRIME having the capability to set these parameters. While this may not reflect actual relationships between these behaviours and diet (i.e. persons with the motivation to improve their diet may also make changes in other health behaviours), our results illustrate the independent effects of dietary changes.

In conclusion, our simulation showed that dietary intakes in the highest quintile of food production-related GHGE are responsible for more deaths than those in the lowest quintile of GHGE, and a shift from these high GHGE diets to low ones would avert about 2 % of deaths per year. This provides additional evidence that diets which are environmentally less impactful are also more healthful and suggests that efforts to implement more environmentally sustainable dietary patterns in the USA could also reduce mortality from CVD.

## Supplementary material

For supplementary material accompanying this paper visit https://doi.org/10.1017/S1368980022000830. click here to view supplementary material

## References

[1] U.S. Global Change Research Program (2017) Climate Science Special Report: Fourth National Climate Assessment, Volume I. Washington, DC: USGCRP. https://purl.fdlp.gov/GPO/gpo86261 (accessed April 2021).

[2] Watts N , Adger WN , Agnolucci P et al. (2015) Health and climate change: policy responses to protect public health. Lancet 386, 1861–1914. 26111439 10.1016/S0140-6736(15)60854-6

[3] Crippa M , Solazzo E , Guizzardi D et al. (2021) Food systems are responsible for a third of global anthropogenic GHG emissions. Nat Food 2,198–209. 37117443 10.1038/s43016-021-00225-9

[4] Wang X , Ouyang Y , Liu J et al. (2014) Fruit and vegetable consumption and mortality from all causes, cardiovascular disease, and cancer: systematic review and dose-response meta-analysis of prospective cohort studies. Br Med J. Published online 29 July 2014. doi: 10.1136/bmj.g4490. PMC4115152 25073782

[5] Tilman D & Clark M (2014) Global diets link environmental sustainability and human health. Nature 515, 518–522. 25383533 10.1038/nature13959

[6] Poore J & Nemecek T (2018) Reducing food’s environmental impacts through producers and consumers. Science 360, 987–992. 29853680 10.1126/science.aaq0216

[7] Heller MC , Willits-Smith A , Meyer R et al. (2018) Greenhouse gas emissions and energy use associated with production of individual self-selected US diets. Environ Res Lett. Published online: 20 March 2018. doi: 10.1088/1748-9326/aab0ac. PMC5964346 29853988

[8] Rose D , Heller MC , Willits-Smith AM et al. (2019) Carbon footprint of self-selected US diets: nutritional, demographic, and behavioral correlates. Am J Clin Nutr 109, 526–534. 30698631 10.1093/ajcn/nqy327 PMC6408204

[9] Payne CL , Scarborough P & Cobiac L (2016) Do low-carbon-emission diets lead to higher nutritional quality and positive health outcomes? A systematic review of the literature. Public Health Nutr 19, 2654–2661. 26975578 10.1017/S1368980016000495 PMC10270842

[10] Vieux F , Soler LG , Touazi D et al. (2013) High nutritional quality is not associated with low greenhouse gas emissions in self-selected diets of French adults. Am J Clin Nutr 97, 569–583. 23364012 10.3945/ajcn.112.035105

[11] Aleksandrowicz L , Green R , Joy EJM et al. (2016) The impacts of dietary change on Greenhouse gas emissions, land use, water use, and health: a systematic review. PLoS One. Published online 3 November 2016. doi: 10.1371/journal.pone.0165797. PMC5094759 27812156

[12] Aston LM , Smith JN & Powles JW (2012) Impact of a reduced red and processed meat dietary pattern on disease risks and greenhouse gas emissions in the UK: a modelling study. BMJ Open. Published online 10 September 2012. doi: 10.1136/bmjopen-2012-001072. PMC3467613 22964113

[13] Soret S , Mejia A , Batech M et al. (2014) Climate change mitigation and health effects of varied dietary patterns in real-life settings throughout North America. Am J Clin Nutr 100, 490s–495s. 24898230 10.3945/ajcn.113.071589

[14] Hallstrom E , Gee Q , Scarborough P et al. (2017) A healthier US diet could reduce greenhouse gas emissions from both the food and health care systems. Clim Change 142, 199–212.

[15] Scarborough P , Harrington RA , Mizdrak A et al. (2014) The preventable risk integrated Model and its use to estimate the health impact of public health policy scenarios. Scientifica. Published online 25 September 2014. doi: 10.1155/2014/748750. PMC4195430 25328757

[16] Filardo G , Pollock BD & Edgerton J (2017) Categorizing body mass index biases assessment of the association with post-coronary artery bypass graft mortality. Eur J Cardio-Thorac 52, 924–929. 10.1093/ejcts/ezx138 28498926

[17] USDA, ARS. FoodData Central. https://fdc.nal.usda.gov/ (accessed October 2021).

[18] Scarborough P , Allender S , Clarke D et al. (2012) Modelling the health impact of environmentally sustainable dietary scenarios in the UK. Eur J Clin Nutr 66, 710–715. 22491494 10.1038/ejcn.2012.34 PMC3389618

[19] Milner J , Green R , Dangour AD et al. (2015) Health effects of adopting low greenhouse gas emission diets in the UK. BMJ Open. Published online 30 April 2015. doi: 10.1136/bmjopen-2014-007364. PMC4420981 25929258

[20] Guenther PM , Casavale KO , Reedy J et al. (2013) Update of the healthy eating index: HEI-2010. J Acad Nutr Diet 113, 569–580. 23415502 10.1016/j.jand.2012.12.016 PMC3810369

[21] Willits-Smith A , Aranda R , Heller MC & Rose D (2020) Addressing the carbon footprint, healthfulness, and costs of self-selected diets in the USA: a population-based cross-sectional study. Lancet Planet Health. Published online March 2020. doi: 10.1016/s2542-5196(20)30055-3. PMC7232940 32220679

[22] Hrynowski Z (2021) What percentage of Americans are vegetarian? Gallup. https://news.gallup.com/poll/267074/percentage-americans-vegetarian.aspx (accessed November 2021).

