# Supplementary material for: Do diets with higher carbon footprints increase the risk of mortality? A population-based simulation study using self-selected diets from the USA
Source: Public Health Nutr. 2022 Mar 31;25(8):2322–8. doi: 10.1017/S1368980022000830 (PMC9991612; doi:10.1017/S1368980022000830)
Supplement: Supplementary file 1 [file S1368980022000830sup.zip › S1368980022000830sup001.docx]

**Supplementary Tables**

**“****Do diets with higher carbon footprints increase the risk of mortality? A population-based simulation study using self-selected diets from the United States”**

**Supplementary Table 1.** **Sex- and age-specific dietary model inputs**

| **Daily dietary components:** | **HiGHGE Diet** | **LoGHGE Diet** |
| --- | --- | --- |
| **Males ages 18-24** |  |  |
| Mean calories | 2400 | 2400 |
| Mean(SD) grams of fruit | 211(355) | 251(398) |
| % consuming <1 fruit portion | 62% | 67% |
| Mean(SD) grams of vegetables | 187(173) | 140(135) * |
| % consuming <1 vegetable portion | 12% | 25% * |
| Mean(SD) grams of fiber | 12.8(7.9) | 15.4(9.9) * |
| Mean(SD) grams of salt | 4.0(1.4) | 3.8(1.3) |
| %(SD) of calories from fat | 32.8(9.5) | 27.9(10.1) ** |
| %(SD) of calories from saturated fat | 11.8(4.3) | 8.2(3.6) *** |
| %(SD) of calories from MUFA | 12.4(3.8) | 10.1(4.4) *** |
| %(SD) of calories from PUFA | 5.3(2.8) | 7.2(3.5) *** |
| Mean(SD) milligrams of cholesterol | 365(197) | 209(169) *** |
|  |  |  |
| **Females ages 18-24** |  |  |
| Mean calories | 2000 | 2000 |
| Mean(SD) grams of fruit | 270(664) | 226(308) |
| % consuming <1 fruit portion | 59% | 57% |
| Mean(SD) grams of vegetables | 170(154) | 149(179) |
| % consuming <1 vegetable portion | 13% | 20% |
| Mean(SD) grams of fiber | 11.4(6.4) | 15.0(9.8) ** |
| Mean(SD) grams of salt | 3.4(1.6) | 3.0(1.1) ** |
| %(SD) of calories from fat | 32.6(10.6) | 30.4(10.6) |
| %(SD) of calories from saturated fat | 11.6(5.1) | 9.2(3.7) *** |
| %(SD) of calories from MUFA | 12.1(4.2) | 10.8(4.1) ** |
| %(SD) of calories from PUFA | 5.8(3.3) | 7.9(4.3) *** |
| Mean(SD) milligrams of cholesterol | 290(191) | 161(126) *** |

HiGHGE and LoGHGE, highest and lowest quintiles of diets ranked on greenhouse gas emissions.

SD, standard deviation. MUFA, monounsaturated fatty acids. PUFA, polyunsaturated fatty acids.

Mean values were significantly different from those of HiGHGE diet group: ******P*<0•05, *******P*<0•01, ********P*<0•001.

**Supplementary Table 1** (**continued)**

| **Daily dietary components:** | **HiGHGE Diet** | **LoGHGE Diet** |
| --- | --- | --- |
| **Males ages 25-39** |  |  |
| Mean calories | 2400 | 2400 |
| Mean(SD) grams of fruit | 206(333) | 214(257) |
| % consuming <1 fruit portion | 66% | 50% ** |
| Mean(SD) grams of vegetables | 221(177) | 188(154) * |
| % consuming <1 vegetable portion | 10% | 11% |
| Mean(SD) grams of fiber | 14.7(8.0) | 19.6(10.6) *** |
| Mean(SD) grams of salt | 4.2(1.4) | 3.6(1.3) *** |
| %(SD) of calories from fat | 34.4(8.5) | 29.6(8.5) *** |
| %(SD) of calories from saturated fat | 12.1(3.7) | 8.5(3.2) *** |
| %(SD) of calories from MUFA | 13.1(3.7) | 10.7(3.7) *** |
| %(SD) of calories from PUFA | 5.7(2.4) | 8.0(3.2) *** |
| Mean(SD) milligrams of cholesterol | 408(229) | 213(140) *** |
|  |  |  |
| **Females ages 25-39** |  |  |
| Mean calories | 1800 | 1800 |
| Mean(SD) grams of fruit | 208(298) | 157(189) |
| % consuming <1 fruit portion | 49% | 51% |
| Mean(SD) grams of vegetables | 226(274) | 179(141) * |
| % consuming <1 vegetable portion | 9% | 14% |
| Mean(SD) grams of fiber | 13.7(8.2) | 15.8(9.1) ** |
| Mean(SD) grams of salt | 3.4(1.2) | 2.7(0.9) *** |
| %(SD) of calories from fat | 35.2(9.8) | 30.5(9.3) *** |
| %(SD) of calories from saturated fat | 12.4(4.1) | 8.4(3.0) *** |
| %(SD) of calories from MUFA | 13.0(4.3) | 11.0(4.3) *** |
| %(SD) of calories from PUFA | 6.3(3.2) | 8.6(3.7) *** |
| Mean(SD) milligrams of cholesterol | 274(170) | 134(102) *** |

HiGHGE and LoGHGE, highest and lowest quintiles of diets ranked on greenhouse gas emissions.

SD, standard deviation. MUFA, monounsaturated fatty acids. PUFA, polyunsaturated fatty acids.

Mean values were significantly different from those of HiGHGE diet group: ******P*<0•05, *******P*<0•01, ********P*<0•001.

**Supplementary Table 1 (continued)**

| **Daily dietary components:** | **HiGHGE Diet** | **LoGHGE Diet** |
| --- | --- | --- |
| **Males ages 40-49** |  |  |
| Mean calories | 2200 | 2200 |
| Mean(SD) grams of fruit | 189(263) | 159(193) |
| % consuming <1 fruit portion | 59% | 55% |
| Mean(SD) grams of vegetables | 203(141) | 179(164) |
| % consuming <1 vegetable portion | 9% | 16% |
| Mean(SD) grams of fiber | 13.5(7.8) | 17.0(9.3) ** |
| Mean(SD) grams of salt | 3.8(1.3) | 3.1(0.9) *** |
| %(SD) of calories from fat | 34.5(8.9) | 31.4(9.9) * |
| %(SD) of calories from saturated fat | 12.1(3.8) | 8.9(3.5) *** |
| %(SD) of calories from MUFA | 13.0(3.5) | 11.6(4.1) ** |
| %(SD) of calories from PUFA | 6.0(2.7) | 8.4(3.6) *** |
| Mean(SD) milligrams of cholesterol | 375(197) | 222(169) *** |
|  |  |  |
| **Females ages 40-49** |  |  |
| Mean calories | 1800 | 1800 |
| Mean(SD) grams of fruit | 159(277) | 142(170) |
| % consuming <1 fruit portion | 59% | 50% |
| Mean(SD) grams of vegetables | 184(139) | 140(116) ** |
| % consuming <1 vegetable portion | 5% | 16%** |
| Mean(SD) grams of fiber | 12.7(5.5) | 15.0(8.2) *** |
| Mean(SD) grams of salt | 3.2(1.0) | 2.7(1.0) *** |
| %(SD) of calories from fat | 35.3(8.1) | 31.7(9.4) ** |
| %(SD) of calories from saturated fat | 12.4(3.7) | 9.2(3.0) *** |
| %(SD) of calories from MUFA | 12.9(3.4) | 11.4(4.0) ** |
| %(SD) of calories from PUFA | 6.6(2.9) | 8.4(3.6) *** |
| Mean(SD) milligrams of cholesterol | 284(134) | 160(112) *** |

HiGHGE and LoGHGE, highest and lowest quintiles of diets ranked on greenhouse gas emissions.

SD, standard deviation. MUFA, monounsaturated fatty acids. PUFA, polyunsaturated fatty acids.

Mean values were significantly different from those of HiGHGE diet group: ******P*<0•05, *******P*<0•01, ********P*<0•001.

**Supplementary Table 1 (continued)**

| **Daily dietary components:** | **HiGHGE Diet** | **LoGHGE Diet** |
| --- | --- | --- |
| **Males ages 50-64** |  |  |
| Mean calories | 2200 | 2200 |
| Mean(SD) grams of fruit | 173(203) | 260(357) ** |
| % consuming <1 fruit portion | 49% | 43% |
| Mean(SD) grams of vegetables | 207(168) | 188(180) |
| % consuming <1 vegetable portion | 12% | 17% |
| Mean(SD) grams of fiber | 15.0(8.1) | 21.1(16.7) *** |
| Mean(SD) grams of salt | 3.7(1.2) | 3.2(1.0) *** |
| %(SD) of calories from fat | 36.3(9.4) | 31.9(10.0) *** |
| %(SD) of calories from saturated fat | 12.3(3.9) | 8.7(3.2) *** |
| %(SD) of calories from MUFA | 13.8(4.1) | 11.7(4.6) *** |
| %(SD) of calories from PUFA | 6.6(3.0) | 8.9(3.9) *** |
| Mean(SD) milligrams of cholesterol | 361(191) | 191(141) *** |
|  |  |  |
| **Females ages 50-64** |  |  |
| Mean calories | 1600 | 1600 |
| Mean(SD) grams of fruit | 181(185) | 151(153) |
| % consuming <1 fruit portion | 44% | 36% |
| Mean(SD) grams of vegetables | 205(162) | 165(154) ** |
| % consuming <1 vegetable portion | 8% | 14% * |
| Mean(SD) grams of fiber | 13.3(6.5) | 15.9(8.4) *** |
| Mean(SD) grams of salt | 3.0(1.0) | 2.4(0.7) *** |
| %(SD) of calories from fat | 35.0(8.2) | 32.8(9.5) * |
| %(SD) of calories from saturated fat | 12.5(3.9) | 8.7(2.9) *** |
| %(SD) of calories from MUFA | 12.8(3.6) | 11.9(4.3) |
| %(SD) of calories from PUFA | 6.3(2.7) | 9.5(4.1) *** |
| Mean(SD) milligrams of cholesterol | 274(163) | 124(98) *** |

HiGHGE and LoGHGE, highest and lowest quintiles of diets ranked on greenhouse gas emissions.

SD, standard deviation. MUFA, monounsaturated fatty acids. PUFA, polyunsaturated fatty acids.

Mean values were significantly different from those of HiGHGE diet group: ******P*<0•05, *******P*<0•01, ********P*<0•001.

**Supplementary Table 2. Sex- and age-specific absolute annual mortality counts by ICD-9 disease categories^1^**

| **Sex/Age** | **I60-I69: Cerebrovascular diseases** | **I20-I25: Ischaemic heart diseases** | **C00-C14: Lip, oral cavity and pharynx** | **C15: Oesophagus** | **C16: Stomach** | **C34: Bronchus and lung** |
| --- | --- | --- | --- | --- | --- | --- |
| **M15-19** | 40 | 27 | 5 | 0 | 0 | 5 |
| **M20-24** | 73 | 80 | 5 | 4 | 5 | 12 |
| **M25-29** | 90 | 246 | 9 | 7 | 28 | 25 |
| **M30-34** | 162 | 563 | 14 | 18 | 32 | 54 |
| **M35-39** | 401 | 1,339 | 48 | 43 | 83 | 177 |
| **M40-44** | 742 | 3,393 | 120 | 164 | 165 | 734 |
| **M45-49** | 1,410 | 7,051 | 289 | 459 | 280 | 2,298 |
| **M50-54** | 2,097 | 11,475 | 568 | 785 | 424 | 4,641 |
| **M55-59** | 2,757 | 15,446 | 761 | 1,287 | 561 | 7,516 |
| **M60-64** | 3,235 | 18,030 | 821 | 1,580 | 698 | 10,810 |
| **M Total** | **11,007** | **57,650** | **2,640** | **4,347** | **2,276** | **26,272** |
|  |  |  |  |  |  |  |
| **F15-19** | 31 | 13 | 3 | 0 | 0 | 1 |
| **F20-24** | 51 | 31 | 5 | 1 | 7 | 7 |
| **F25-29** | 90 | 83 | 4 | 0 | 18 | 15 |
| **F30-34** | 163 | 156 | 17 | 3 | 49 | 41 |
| **F35-39** | 332 | 447 | 21 | 13 | 92 | 175 |
| **F40-44** | 658 | 1,040 | 62 | 26 | 116 | 766 |
| **F45-49** | 1,206 | 2,230 | 102 | 84 | 212 | 2,055 |
| **F50-54** | 1,672 | 3,634 | 177 | 124 | 235 | 3,486 |
| **F55-59** | 1,988 | 5,287 | 224 | 229 | 280 | 5,111 |
| **F60-64** | 2,520 | 7,401 | 213 | 283 | 340 | 7,779 |
| **F Total** | **8,711** | **20,322** | **828** | **763** | **1,349** | **19,436** |

^1^Mortality values came from *wonder.cdc.gov*. While PRIME includes population mortality for a wide variety of non-communicable diseases, not all were affected by the baseline and counterfactual diet parameters included in this study. This table includes all the inputs for mortality data in the model. Of the cancer types modeled in PRIME, only lung and colorectal cancers showed any deaths averted in our scenarios. For more information on how PRIME works, see Scarborough et al 2014.^15^

**Supplementary Table 2 (continued)**

| **Sex/Age** | **C25: Pancreas** | **C18-20: Colorectum** | **C50: Breast** | **C54.1: Endometrium** | **C64: Kidney** | **C67: Bladder** | **I10-I15: Hypertensive disease** | **E11,E14: Diabetes** |
| --- | --- | --- | --- | --- | --- | --- | --- | --- |
| **M15-19** | 0 | 9 | 0 | 0 | 7 | 0 | 5 | 31 |
| **M20-24** | 3 | 17 | 0 | 0 | 13 | 0 | 33 | 67 |
| **M25-29** | 6 | 58 | 0 | 0 | 6 | 3 | 88 | 123 |
| **M30-34** | 26 | 97 | 0 | 0 | 26 | 1 | 166 | 236 |
| **M35-39** | 82 | 225 | 4 | 0 | 45 | 18 | 343 | 479 |
| **M40-44** | 243 | 480 | 6 | 0 | 116 | 39 | 666 | 749 |
| **M45-49** | 541 | 982 | 12 | 0 | 331 | 131 | 1,126 | 1,376 |
| **M50-54** | 1,149 | 1,675 | 24 | 0 | 554 | 275 | 1,467 | 2,137 |
| **M55-59** | 1,705 | 2,367 | 30 | 0 | 810 | 478 | 1,621 | 3,042 |
| **M60-64** | 2,167 | 2,972 | 40 | 0 | 1,011 | 698 | 1,566 | 3,602 |
| **M Total** | **5,922** | **8,882** | **116** | **0** | **2,919** | **1,643** | **7,081** | **11,842** |
|  |  |  |  |  |  |  |  |  |
| **F15-19** | 1 | 4 | 1 | 1 | 4 | 0 | 1 | 24 |
| **F20-24** | 1 | 5 | 14 | 1 | 10 | 0 | 10 | 46 |
| **F25-29** | 4 | 38 | 65 | 10 | 9 | 1 | 37 | 92 |
| **F30-34** | 16 | 82 | 279 | 21 | 9 | 2 | 79 | 159 |
| **F35-39** | 50 | 181 | 736 | 62 | 21 | 6 | 130 | 270 |
| **F40-44** | 163 | 416 | 1,438 | 106 | 58 | 30 | 310 | 486 |
| **F45-49** | 386 | 840 | 2,494 | 210 | 143 | 69 | 507 | 785 |
| **F50-54** | 732 | 1,296 | 3,460 | 374 | 215 | 95 | 682 | 1,455 |
| **F55-59** | 1,136 | 1,722 | 4,238 | 668 | 346 | 168 | 792 | 2,043 |
| **F60-64** | 1,499 | 1,997 | 4,448 | 915 | 417 | 220 | 801 | 2,617 |
| **F Total** | **3,988** | **6,581** | **17,173** | **2,368** | **1,232** | **591** | **3,349** | **7,977** |

^1^Mortality values came from *wonder.cdc.gov*. While PRIME includes population mortality for a wide variety of non-communicable diseases, not all were affected by the baseline and counterfactual diet parameters included in this study. This table includes all the inputs for mortality data in the model. Of the cancer types modeled in PRIME, only lung and colorectal cancers showed any deaths averted in our scenarios. For more information on how PRIME works, see Scarborough et al 2014.^15^

**Supplementary Table 2 (continued)**

| **Sex/Age** | **C22: Liver** | **C53: Cervix** | **J40-J44: COPD** | **K70, K74: Liver disease** | **I50: Heart failure** | **I71: Aortic aneurysm** | **I05-09: Rheumatic heart disease** | **N18: Renal failure** | **TOTAL** |
| --- | --- | --- | --- | --- | --- | --- | --- | --- | --- |
| **M15-19** | 7 | 0 | 43 | 2 | 10 | 11 | 2 | 7 | **211** |
| **M20-24** | 10 | 0 | 53 | 12 | 15 | 21 | 4 | 30 | **457** |
| **M25-29** | 20 | 0 | 76 | 68 | 15 | 22 | 7 | 50 | **947** |
| **M30-34** | 38 | 0 | 74 | 193 | 39 | 52 | 7 | 75 | **1,873** |
| **M35-39** | 75 | 0 | 108 | 492 | 63 | 110 | 13 | 144 | **4,292** |
| **M40-44** | 183 | 0 | 284 | 1,230 | 144 | 168 | 14 | 256 | **9,896** |
| **M45-49** | 560 | 0 | 703 | 2,429 | 248 | 226 | 33 | 448 | **20,933** |
| **M50-54** | 1,409 | 0 | 1,422 | 3,359 | 407 | 341 | 45 | 726 | **34,980** |
| **M55-59** | 1,792 | 0 | 2,478 | 3,278 | 716 | 453 | 56 | 1,053 | **48,207** |
| **M60-64** | 1,449 | 0 | 4,255 | 2,454 | 927 | 640 | 77 | 1,283 | **58,315** |
| **M Total** | **5,543** | **0** | **9,496** | **13,517** | **2,584** | **2,044** | **258** | **4,072** | **180,111** |
|  |  |  |  |  |  |  |  |  |  |
| **F15-19** | 6 | 1 | 28 | 1 | 2 | 1 | 2 | 12 | **137** |
| **F20-24** | 15 | 7 | 25 | 15 | 16 | 5 | 4 | 28 | **304** |
| **F25-29** | 13 | 50 | 51 | 43 | 12 | 7 | 8 | 42 | **692** |
| **F30-34** | 19 | 133 | 62 | 80 | 21 | 18 | 6 | 70 | **1,485** |
| **F35-39** | 37 | 278 | 120 | 250 | 42 | 30 | 11 | 115 | **3,419** |
| **F40-44** | 73 | 367 | 284 | 598 | 68 | 56 | 32 | 181 | **7,334** |
| **F45-49** | 212 | 409 | 694 | 1,111 | 135 | 78 | 48 | 365 | **14,375** |
| **F50-54** | 322 | 506 | 1,334 | 1,313 | 283 | 107 | 51 | 552 | **22,105** |
| **F55-59** | 457 | 490 | 2,163 | 1,207 | 423 | 157 | 88 | 833 | **30,050** |
| **F60-64** | 483 | 402 | 3,881 | 1,065 | 692 | 233 | 116 | 1,036 | **39,358** |
| **F Total** | **1,637** | **2,643** | **8,642** | **5,683** | **1,694** | **692** | **366** | **3,234** | **119,259** |

^1^Mortality values came from *wonder.cdc.gov*. While PRIME includes population mortality for a wide variety of non-communicable diseases, not all were affected by the baseline and counterfactual diet parameters included in this study. This table includes all the inputs for mortality data in the model. Of the cancer types modeled in PRIME, only lung and colorectal cancers showed any deaths averted in our scenarios. For more information on how PRIME works, see Scarborough et al 2014.^15^

**Supplementary Table 3.** **Sex- and age-specific absolute US population in 2007**

| **Age** | **Male** | **Female** |
| --- | --- | --- |
| **15-19** | 11,336,490 | 11,336,490 |
| **20-24** | 10,807,836 | 10,807,836 |
| **25-29** | 10,313,439 | 10,229,259 |
| **30-34** | 9,611,431 | 9,559,334 |
| **35-39** | 10,461,052 | 10,502,839 |
| **40-44** | 10,833,176 | 10,999,163 |
| **45-49** | 11,257,548 | 11,542,213 |
| **50-54** | 10,344,429 | 10,795,749 |
| **55-59** | 8,945,299 | 9,509,473 |
| **60-64** | 7,034,464 | 7,639,198 |
| **Total** | **100,945,164** | **102,921,554** |

Source: wonder.cdc.gov
