# Supplementary material for: Do diets with higher carbon footprints increase the risk of mortality? A population-based simulation study using self-selected diets from the USA
Source: Public Health Nutr. 2022 Mar 31;25(8):2322–8. doi: 10.1017/S1368980022000830 (PMC9991612; doi:10.1017/S1368980022000830)
Supplement: Supplementary file 1 [file S1368980022000830sup.zip › S1368980022000830sup002.docx]

**Supplementary Tables**

**“****Do diets with higher carbon footprints increase the risk of mortality? A population-based simulation study using self-selected diets from the United States”**

**Supplemental Table 1.** **Sex- and age-specific energy intakes* used in PRIME**

|  | **18-24 years** | **25-39 years** | **40-49 years** | **50-64 years** |
| --- | --- | --- | --- | --- |
| **Males** | 2400 | 2400 | 2200 | 2200 |
| **Females** | 2000 | 1800 | 1800 | 1600 |

*Estimated energy intakes at a sedentary activity level came from Appendix 2 of the 2015 Dietary Guidelines for Americans. These estimates use reference values (men at 5 feet 10 inches in height and 154 pounds, women at 5 feet 4 inches tall and 126 pounds) all with estimated energy requirement (EER) equations from the DRI. For further information, see the following sources:

U.S. Department of Health and Human Services and U.S. Department of Agriculture. 2015–2020 Dietary Guidelines for Americans. 8th Edition. December 2015. Available at http://health.gov/dietaryguidelines/2015/guidelines/.

Institute of Medicine. Dietary Reference Intakes for Energy, Carbohydrate, Fiber, Fat, Fatty Acids, Cholesterol, Protein, and Amino Acids. Washington (DC): The National Academies Press; 2002.

**Supplemental Table 2.** **Sex- and age-specific dietary model inputs**

| **Daily dietary components:** | **HiGHGE Diet** | **LoGHGE Diet** |
| --- | --- | --- |
| **Males ages 18-24** |  |  |
| Kilocalories | 2400 | 2400 |
| Mean(SD) grams of fruit | 211(355) | 251(398) |
| % consuming <1 fruit portion | 62% | 67% |
| Mean(SD) grams of vegetables | 187(173) | 140(135) * |
| % consuming <1 vegetable portion | 12% | 25% * |
| Mean(SD) grams of fiber | 12.8(7.9) | 15.4(9.9) * |
| Mean(SD) grams of salt | 4.0(1.4) | 3.8(1.3) |
| %(SD) of calories from fat | 32.8(9.5) | 27.9(10.1) ** |
| %(SD) of calories from saturated fat | 11.8(4.3) | 8.2(3.6) *** |
| %(SD) of calories from MUFA | 12.4(3.8) | 10.1(4.4) *** |
| %(SD) of calories from PUFA | 5.3(2.8) | 7.2(3.5) *** |
| Mean(SD) milligrams of cholesterol | 365(197) | 209(169) *** |
|  |  |  |
| **Females ages 18-24** |  |  |
| Kilocalories | 2000 | 2000 |
| Mean(SD) grams of fruit | 270(664) | 226(308) |
| % consuming <1 fruit portion | 59% | 57% |
| Mean(SD) grams of vegetables | 170(154) | 149(179) |
| % consuming <1 vegetable portion | 13% | 20% |
| Mean(SD) grams of fiber | 11.4(6.4) | 15.0(9.8) ** |
| Mean(SD) grams of salt | 3.4(1.6) | 3.0(1.1) ** |
| %(SD) of calories from fat | 32.6(10.6) | 30.4(10.6) |
| %(SD) of calories from saturated fat | 11.6(5.1) | 9.2(3.7) *** |
| %(SD) of calories from MUFA | 12.1(4.2) | 10.8(4.1) ** |
| %(SD) of calories from PUFA | 5.8(3.3) | 7.9(4.3) *** |
| Mean(SD) milligrams of cholesterol | 290(191) | 161(126) *** |

HiGHGE and LoGHGE, highest and lowest quintiles of diets ranked on greenhouse gas emissions.

SD, standard deviation. MUFA, monounsaturated fatty acids. PUFA, polyunsaturated fatty acids.

Mean values were significantly different from those of HiGHGE diet group: ******P*<0•05, *******P*<0•01, ********P*<0•001.

**Supplemental Table 2** (**continued)**

| **Daily dietary components:** | **HiGHGE Diet** | **LoGHGE Diet** |
| --- | --- | --- |
| **Males ages 25-39** |  |  |
| Kilocalories | 2400 | 2400 |
| Mean(SD) grams of fruit | 206(333) | 214(257) |
| % consuming <1 fruit portion | 66% | 50% ** |
| Mean(SD) grams of vegetables | 221(177) | 188(154) * |
| % consuming <1 vegetable portion | 10% | 11% |
| Mean(SD) grams of fiber | 14.7(8.0) | 19.6(10.6) *** |
| Mean(SD) grams of salt | 4.2(1.4) | 3.6(1.3) *** |
| %(SD) of calories from fat | 34.4(8.5) | 29.6(8.5) *** |
| %(SD) of calories from saturated fat | 12.1(3.7) | 8.5(3.2) *** |
| %(SD) of calories from MUFA | 13.1(3.7) | 10.7(3.7) *** |
| %(SD) of calories from PUFA | 5.7(2.4) | 8.0(3.2) *** |
| Mean(SD) milligrams of cholesterol | 408(229) | 213(140) *** |
|  |  |  |
| **Females ages 25-39** |  |  |
| Kilocalories | 1800 | 1800 |
| Mean(SD) grams of fruit | 208(298) | 157(189) |
| % consuming <1 fruit portion | 49% | 51% |
| Mean(SD) grams of vegetables | 226(274) | 179(141) * |
| % consuming <1 vegetable portion | 9% | 14% |
| Mean(SD) grams of fiber | 13.7(8.2) | 15.8(9.1) ** |
| Mean(SD) grams of salt | 3.4(1.2) | 2.7(0.9) *** |
| %(SD) of calories from fat | 35.2(9.8) | 30.5(9.3) *** |
| %(SD) of calories from saturated fat | 12.4(4.1) | 8.4(3.0) *** |
| %(SD) of calories from MUFA | 13.0(4.3) | 11.0(4.3) *** |
| %(SD) of calories from PUFA | 6.3(3.2) | 8.6(3.7) *** |
| Mean(SD) milligrams of cholesterol | 274(170) | 134(102) *** |

HiGHGE and LoGHGE, highest and lowest quintiles of diets ranked on greenhouse gas emissions.

SD, standard deviation. MUFA, monounsaturated fatty acids. PUFA, polyunsaturated fatty acids.

Mean values were significantly different from those of HiGHGE diet group: ******P*<0•05, *******P*<0•01, ********P*<0•001.

**Supplemental Table 2 (continued)**

| **Daily dietary components:** | **HiGHGE Diet** | **LoGHGE Diet** |
| --- | --- | --- |
| **Males ages 40-49** |  |  |
| Kilocalories | 2200 | 2200 |
| Mean(SD) grams of fruit | 189(263) | 159(193) |
| % consuming <1 fruit portion | 59% | 55% |
| Mean(SD) grams of vegetables | 203(141) | 179(164) |
| % consuming <1 vegetable portion | 9% | 16% |
| Mean(SD) grams of fiber | 13.5(7.8) | 17.0(9.3) ** |
| Mean(SD) grams of salt | 3.8(1.3) | 3.1(0.9) *** |
| %(SD) of calories from fat | 34.5(8.9) | 31.4(9.9) * |
| %(SD) of calories from saturated fat | 12.1(3.8) | 8.9(3.5) *** |
| %(SD) of calories from MUFA | 13.0(3.5) | 11.6(4.1) ** |
| %(SD) of calories from PUFA | 6.0(2.7) | 8.4(3.6) *** |
| Mean(SD) milligrams of cholesterol | 375(197) | 222(169) *** |
|  |  |  |
| **Females ages 40-49** |  |  |
| Kilocalories | 1800 | 1800 |
| Mean(SD) grams of fruit | 159(277) | 142(170) |
| % consuming <1 fruit portion | 59% | 50% |
| Mean(SD) grams of vegetables | 184(139) | 140(116) ** |
| % consuming <1 vegetable portion | 5% | 16%** |
| Mean(SD) grams of fiber | 12.7(5.5) | 15.0(8.2) *** |
| Mean(SD) grams of salt | 3.2(1.0) | 2.7(1.0) *** |
| %(SD) of calories from fat | 35.3(8.1) | 31.7(9.4) ** |
| %(SD) of calories from saturated fat | 12.4(3.7) | 9.2(3.0) *** |
| %(SD) of calories from MUFA | 12.9(3.4) | 11.4(4.0) ** |
| %(SD) of calories from PUFA | 6.6(2.9) | 8.4(3.6) *** |
| Mean(SD) milligrams of cholesterol | 284(134) | 160(112) *** |

HiGHGE and LoGHGE, highest and lowest quintiles of diets ranked on greenhouse gas emissions.

SD, standard deviation. MUFA, monounsaturated fatty acids. PUFA, polyunsaturated fatty acids.

Mean values were significantly different from those of HiGHGE diet group: ******P*<0•05, *******P*<0•01, ********P*<0•001.

**Supplemental Table 2 (continued)**

| **Daily dietary components:** | **HiGHGE Diet** | **LoGHGE Diet** |
| --- | --- | --- |
| **Males ages 50-64** |  |  |
| Kilocalories | 2200 | 2200 |
| Mean(SD) grams of fruit | 173(203) | 260(357) ** |
| % consuming <1 fruit portion | 49% | 43% |
| Mean(SD) grams of vegetables | 207(168) | 188(180) |
| % consuming <1 vegetable portion | 12% | 17% |
| Mean(SD) grams of fiber | 15.0(8.1) | 21.1(16.7) *** |
| Mean(SD) grams of salt | 3.7(1.2) | 3.2(1.0) *** |
| %(SD) of calories from fat | 36.3(9.4) | 31.9(10.0) *** |
| %(SD) of calories from saturated fat | 12.3(3.9) | 8.7(3.2) *** |
| %(SD) of calories from MUFA | 13.8(4.1) | 11.7(4.6) *** |
| %(SD) of calories from PUFA | 6.6(3.0) | 8.9(3.9) *** |
| Mean(SD) milligrams of cholesterol | 361(191) | 191(141) *** |
|  |  |  |
| **Females ages 50-64** |  |  |
| Kilocalories | 1600 | 1600 |
| Mean(SD) grams of fruit | 181(185) | 151(153) |
| % consuming <1 fruit portion | 44% | 36% |
| Mean(SD) grams of vegetables | 205(162) | 165(154) ** |
| % consuming <1 vegetable portion | 8% | 14% * |
| Mean(SD) grams of fiber | 13.3(6.5) | 15.9(8.4) *** |
| Mean(SD) grams of salt | 3.0(1.0) | 2.4(0.7) *** |
| %(SD) of calories from fat | 35.0(8.2) | 32.8(9.5) * |
| %(SD) of calories from saturated fat | 12.5(3.9) | 8.7(2.9) *** |
| %(SD) of calories from MUFA | 12.8(3.6) | 11.9(4.3) |
| %(SD) of calories from PUFA | 6.3(2.7) | 9.5(4.1) *** |
| Mean(SD) milligrams of cholesterol | 274(163) | 124(98) *** |

HiGHGE and LoGHGE, highest and lowest quintiles of diets ranked on greenhouse gas emissions.

SD, standard deviation. MUFA, monounsaturated fatty acids. PUFA, polyunsaturated fatty acids.

Mean values were significantly different from those of HiGHGE diet group: ******P*<0•05, *******P*<0•01, ********P*<0•001.

**Supplemental Table 3. Sex- and age-specific absolute annual mortality counts by ICD-9 disease categories^1^**

| **Sex/Age** | **I60-I69: Cerebrovascular diseases** | **I20-I25: Ischaemic heart diseases** | **C00-C14: Lip, oral cavity and pharynx** | **C15: Oesophagus** | **C16: Stomach** | **C34: Bronchus and lung** |
| --- | --- | --- | --- | --- | --- | --- |
| **M15-19** | 40 | 27 | 5 | 0 | 0 | 5 |
| **M20-24** | 73 | 80 | 5 | 4 | 5 | 12 |
| **M25-29** | 90 | 246 | 9 | 7 | 28 | 25 |
| **M30-34** | 162 | 563 | 14 | 18 | 32 | 54 |
| **M35-39** | 401 | 1,339 | 48 | 43 | 83 | 177 |
| **M40-44** | 742 | 3,393 | 120 | 164 | 165 | 734 |
| **M45-49** | 1,410 | 7,051 | 289 | 459 | 280 | 2,298 |
| **M50-54** | 2,097 | 11,475 | 568 | 785 | 424 | 4,641 |
| **M55-59** | 2,757 | 15,446 | 761 | 1,287 | 561 | 7,516 |
| **M60-64** | 3,235 | 18,030 | 821 | 1,580 | 698 | 10,810 |
| **M Total** | **11,007** | **57,650** | **2,640** | **4,347** | **2,276** | **26,272** |
|  |  |  |  |  |  |  |
| **F15-19** | 31 | 13 | 3 | 0 | 0 | 1 |
| **F20-24** | 51 | 31 | 5 | 1 | 7 | 7 |
| **F25-29** | 90 | 83 | 4 | 0 | 18 | 15 |
| **F30-34** | 163 | 156 | 17 | 3 | 49 | 41 |
| **F35-39** | 332 | 447 | 21 | 13 | 92 | 175 |
| **F40-44** | 658 | 1,040 | 62 | 26 | 116 | 766 |
| **F45-49** | 1,206 | 2,230 | 102 | 84 | 212 | 2,055 |
| **F50-54** | 1,672 | 3,634 | 177 | 124 | 235 | 3,486 |
| **F55-59** | 1,988 | 5,287 | 224 | 229 | 280 | 5,111 |
| **F60-64** | 2,520 | 7,401 | 213 | 283 | 340 | 7,779 |
| **F Total** | **8,711** | **20,322** | **828** | **763** | **1,349** | **19,436** |

^1^Mortality values came from *wonder.cdc.gov*. While PRIME includes population mortality for a wide variety of non-communicable diseases, not all were affected by the baseline and counterfactual diet parameters included in this study. This table includes all the inputs for mortality data in the model. Of the cancer types modeled in PRIME, only lung and colorectal cancers showed any deaths averted in our scenarios. For more information on how PRIME works, see Scarborough et al 2014.^15^

**Supplemental Table 3 (continued)**

| **Sex/Age** | **C25: Pancreas** | **C18-20: Colorectum** | **C50: Breast** | **C54.1: Endometrium** | **C64: Kidney** | **C67: Bladder** | **I10-I15: Hypertensive disease** | **E11,E14: Diabetes** |
| --- | --- | --- | --- | --- | --- | --- | --- | --- |
| **M15-19** | 0 | 9 | 0 | 0 | 7 | 0 | 5 | 31 |
| **M20-24** | 3 | 17 | 0 | 0 | 13 | 0 | 33 | 67 |
| **M25-29** | 6 | 58 | 0 | 0 | 6 | 3 | 88 | 123 |
| **M30-34** | 26 | 97 | 0 | 0 | 26 | 1 | 166 | 236 |
| **M35-39** | 82 | 225 | 4 | 0 | 45 | 18 | 343 | 479 |
| **M40-44** | 243 | 480 | 6 | 0 | 116 | 39 | 666 | 749 |
| **M45-49** | 541 | 982 | 12 | 0 | 331 | 131 | 1,126 | 1,376 |
| **M50-54** | 1,149 | 1,675 | 24 | 0 | 554 | 275 | 1,467 | 2,137 |
| **M55-59** | 1,705 | 2,367 | 30 | 0 | 810 | 478 | 1,621 | 3,042 |
| **M60-64** | 2,167 | 2,972 | 40 | 0 | 1,011 | 698 | 1,566 | 3,602 |
| **M Total** | **5,922** | **8,882** | **116** | **0** | **2,919** | **1,643** | **7,081** | **11,842** |
|  |  |  |  |  |  |  |  |  |
| **F15-19** | 1 | 4 | 1 | 1 | 4 | 0 | 1 | 24 |
| **F20-24** | 1 | 5 | 14 | 1 | 10 | 0 | 10 | 46 |
| **F25-29** | 4 | 38 | 65 | 10 | 9 | 1 | 37 | 92 |
| **F30-34** | 16 | 82 | 279 | 21 | 9 | 2 | 79 | 159 |
| **F35-39** | 50 | 181 | 736 | 62 | 21 | 6 | 130 | 270 |
| **F40-44** | 163 | 416 | 1,438 | 106 | 58 | 30 | 310 | 486 |
| **F45-49** | 386 | 840 | 2,494 | 210 | 143 | 69 | 507 | 785 |
| **F50-54** | 732 | 1,296 | 3,460 | 374 | 215 | 95 | 682 | 1,455 |
| **F55-59** | 1,136 | 1,722 | 4,238 | 668 | 346 | 168 | 792 | 2,043 |
| **F60-64** | 1,499 | 1,997 | 4,448 | 915 | 417 | 220 | 801 | 2,617 |
| **F Total** | **3,988** | **6,581** | **17,173** | **2,368** | **1,232** | **591** | **3,349** | **7,977** |

^1^Mortality values came from *wonder.cdc.gov*. While PRIME includes population mortality for a wide variety of non-communicable diseases, not all were affected by the baseline and counterfactual diet parameters included in this study. This table includes all the inputs for mortality data in the model. Of the cancer types modeled in PRIME, only lung and colorectal cancers showed any deaths averted in our scenarios. For more information on how PRIME works, see Scarborough et al 2014.^15^

**Supplemental Table 3 (continued)**

| **Sex/Age** | **C22: Liver** | **C53: Cervix** | **J40-J44: COPD** | **K70, K74: Liver disease** | **I50: Heart failure** | **I71: Aortic aneurysm** | **I05-09: Rheumatic heart disease** | **N18: Renal failure** | **TOTAL** |
| --- | --- | --- | --- | --- | --- | --- | --- | --- | --- |
| **M15-19** | 7 | 0 | 43 | 2 | 10 | 11 | 2 | 7 | **211** |
| **M20-24** | 10 | 0 | 53 | 12 | 15 | 21 | 4 | 30 | **457** |
| **M25-29** | 20 | 0 | 76 | 68 | 15 | 22 | 7 | 50 | **947** |
| **M30-34** | 38 | 0 | 74 | 193 | 39 | 52 | 7 | 75 | **1,873** |
| **M35-39** | 75 | 0 | 108 | 492 | 63 | 110 | 13 | 144 | **4,292** |
| **M40-44** | 183 | 0 | 284 | 1,230 | 144 | 168 | 14 | 256 | **9,896** |
| **M45-49** | 560 | 0 | 703 | 2,429 | 248 | 226 | 33 | 448 | **20,933** |
| **M50-54** | 1,409 | 0 | 1,422 | 3,359 | 407 | 341 | 45 | 726 | **34,980** |
| **M55-59** | 1,792 | 0 | 2,478 | 3,278 | 716 | 453 | 56 | 1,053 | **48,207** |
| **M60-64** | 1,449 | 0 | 4,255 | 2,454 | 927 | 640 | 77 | 1,283 | **58,315** |
| **M Total** | **5,543** | **0** | **9,496** | **13,517** | **2,584** | **2,044** | **258** | **4,072** | **180,111** |
|  |  |  |  |  |  |  |  |  |  |
| **F15-19** | 6 | 1 | 28 | 1 | 2 | 1 | 2 | 12 | **137** |
| **F20-24** | 15 | 7 | 25 | 15 | 16 | 5 | 4 | 28 | **304** |
| **F25-29** | 13 | 50 | 51 | 43 | 12 | 7 | 8 | 42 | **692** |
| **F30-34** | 19 | 133 | 62 | 80 | 21 | 18 | 6 | 70 | **1,485** |
| **F35-39** | 37 | 278 | 120 | 250 | 42 | 30 | 11 | 115 | **3,419** |
| **F40-44** | 73 | 367 | 284 | 598 | 68 | 56 | 32 | 181 | **7,334** |
| **F45-49** | 212 | 409 | 694 | 1,111 | 135 | 78 | 48 | 365 | **14,375** |
| **F50-54** | 322 | 506 | 1,334 | 1,313 | 283 | 107 | 51 | 552 | **22,105** |
| **F55-59** | 457 | 490 | 2,163 | 1,207 | 423 | 157 | 88 | 833 | **30,050** |
| **F60-64** | 483 | 402 | 3,881 | 1,065 | 692 | 233 | 116 | 1,036 | **39,358** |
| **F Total** | **1,637** | **2,643** | **8,642** | **5,683** | **1,694** | **692** | **366** | **3,234** | **119,259** |

^1^Mortality values came from *wonder.cdc.gov*. While PRIME includes population mortality for a wide variety of non-communicable diseases, not all were affected by the baseline and counterfactual diet parameters included in this study. This table includes all the inputs for mortality data in the model. Of the cancer types modeled in PRIME, only lung and colorectal cancers showed any deaths averted in our scenarios. For more information on how PRIME works, see Scarborough et al 2014.^15^

**Supplemental Table 4.** **Sex- and age-specific absolute US population in 2007**

| **Age** | **Male** | **Female** |
| --- | --- | --- |
| **15-19** | 11,336,490 | 11,336,490 |
| **20-24** | 10,807,836 | 10,807,836 |
| **25-29** | 10,313,439 | 10,229,259 |
| **30-34** | 9,611,431 | 9,559,334 |
| **35-39** | 10,461,052 | 10,502,839 |
| **40-44** | 10,833,176 | 10,999,163 |
| **45-49** | 11,257,548 | 11,542,213 |
| **50-54** | 10,344,429 | 10,795,749 |
| **55-59** | 8,945,299 | 9,509,473 |
| **60-64** | 7,034,464 | 7,639,198 |
| **Total** | **100,945,164** | **102,921,554** |

Source: wonder.cdc.gov
